# Correlation between relative dose intensity of adjuvant S-1 chemotherapy and psoas muscle mass volume and survival after resection of pancreatic ductal adenocarcinoma: A retrospective study

**DOI:** 10.1097/MD.0000000000038292

**Published:** 2024-05-24

**Authors:** Teruhisa Sakamoto, Mikiya Kishino, Yuki Murakami, Kozo Miyatani, Takehiko Hanaki, Yuji Shishido, Kyoichi Kihara, Tomoyuki Matsunaga, Manabu Yamamoto, Naruo Tokuyasu, Yoshiyuki Fujiwara

**Affiliations:** a Division of Gastrointestinal and Pediatric Surgery, Department of Surgery, Faculty of Medicine, Tottori University, Yonago, Japan.

**Keywords:** adjuvant chemotherapy, pancreatic cancer, psoas muscle mass, relative dose intensity, S-1

## Abstract

This study aimed to investigate the prognostic relationship between relative dose intensity (RDI) of adjuvant S-1 chemotherapy and psoas muscle mass volume (PMV) in patients with resected pancreatic ductal adenocarcinoma. We enrolled 105 patients with histologically confirmed pancreatic ductal adenocarcinoma who had undergone pancreatectomy. Adjuvant S-1 chemotherapy was administered to 72 (68.6%) of the 105 patients and not to the remaining 33 patients. Patients who received adjuvant S-1 chemotherapy were stratified into high- and low-RDI groups by the cutoff value for RDI. Five-year overall survival (OS) and relapse-free survival (RFS) rates were significantly higher in the high- than in the low-RDI group. Similarly, both the 5-year OS and RFS rates were significantly greater among patients in the high-PMV group than among patients in the low-PMV group. The RDI was an independent prognostic factor in our study patients. Furthermore, patients who received adjuvant S-1 chemotherapy were stratified into 3 groups: those with both high RDI and high-PMV, Group A; those with either high RDI or high PMV (but not both), Group B; and those with both low RDI and low-PMV, group C. There were statistically significant differences in 5-year OS and RFS between 3 patient groups (5-year overall survival: *P* = .023, 5-year relapse-free survival: *P* = .001). The area under the curve for the combination of RDI and PMV (0.674) was greater than that for RDI alone (0.645). A sufficient dosage of adjuvant S-1 chemotherapy is important in improving survival of patients with resected pancreatic ductal adenocarcinoma. A combination of RDI and PMV may predict the prognosis of patients with resected pancreatic ductal adenocarcinoma more effective than RDI alone.

## 1. Introduction

The incidence of pancreatic cancer has been increasing despite its dismal prognosis, the 5-year overall survival (OS) rate from the time of diagnosis being approximately 10%.^[1,2]^ In Japan, pancreatic cancer is the fourth leading cause of cancer-related death.^[3]^ Surgical resection is essential to overcome this highly lethal cancer. However, even after curative-intent surgery has been achieved, pancreatic ductal adenocarcinoma (PDAC) recurs in approximately 80% of patients.^[4]^ After postoperative recurrence, the prognosis of patients with PADC is extremely poor, the median OS being approximately 7 months.^[5]^ Therefore, various randomized clinical trials on perioperative treatments have been undertaken with the aim of improving the 5-year OS of patients with PDAC who have undergone curative resection.

Postoperative adjuvant chemotherapy has been shown to prolong survival after resection of PADC. On the basis of the results of the ESPAC-04 and PRODIGE24/CCTG trials, modified FOLFIRINOX or gemcitabine with capecitabine is recommended as adjuvant chemotherapy after resection of PDAC in Western countries.^[6–8]^ In Japan, adjuvant S-1 chemotherapy is the standard treatment after resection of PDAC, in accordance with the results of the JASPAC-01 trial, which was a phase 3 study of postoperative adjuvant chemotherapy with gemcitabine monotherapy versus S-1 monotherapy in patients who had undergone resection of PDAC.^[9]^ In this trial, the 5-year survival rate and median survival time of patients who had received sufficient S-1 were reportedly 44.1% and 46.5 months, respectively. Thus, a sufficient S-1 dosage has been found to achieve a survival benefit after surgery for PDAC; however, the prognostic effect of an insufficient S-1 dosage in patients with PDAC has rarely been reported. Furthermore, there is reportedly a close prognostic relationship between relative dose intensity (RDI) of chemotherapy and skeletal muscle mass in patients with gastric cancer.^[10,11]^ However, the prognostic relationship between RDI of adjuvant S-1 chemotherapy and skeletal muscle mass after resection of PDAC has not yet been determined.

In the current study, we investigated the prognostic impact of sufficient and insufficient S-1 dosage administered as postoperative adjuvant chemotherapy after resection of PDAC. We also examined the effects on survival of the relationship between RDI and skeletal muscle mass volume, specifically psoas muscle mass volume (PMV), after resection of PDAC.

## 2. Methods

### 2.1. Patients

We retrospectively reviewed data of 136 patients with histologically confirmed PDAC who had undergone pancreatectomy in our hospital between January 2012 and December 2021. Of these 136 patients, 31 were excluded because of early recurrence (within 6 months after pancreatectomy). Finally, 105 patients were enrolled in this study. Their resectability statuses were as follows: resectable in 101 patients, borderline resectable in 2, and unresectable locally advanced in 2 who had undergone conversion surgery. No study patient had distant metastases. The patients’ clinicopathological findings were collected from their medical records and the histopathological findings were classified in accordance with the 8th edition of the International Union Against Cancer Tumor-Node-Metastasis classification system.^[12]^

All patients in this study were of Japanese ethnicity.

The Tottori University Hospital Ethics Committee approved this study (No. 22A157) and the requirement for informed consent was waived.

### 2.2. Treatment

#### 2.2.1. Adjuvant S-1 chemotherapy

In Japan, in accordance with the results of the JASPAC01 trial, adjuvant chemotherapy with S-1 within 10 weeks after pancreatectomy is recommended for patients who have undergone curative surgery for pancreatic cancer. The S-1 dose is based on the body surface area (BSA) as follows: BSA < 1.25 m^2^, 80 mg/day; BSA 1.25–1.5 m^2^, 100 mg/day; BSA > 1.50 m^2^, 120 mg/day. The patients received S-1 for 28 consecutive days followed by a 14-day rest at 6-week intervals for 6 months. The dose of S-1 was reduced by 20 mg/day in patients with impaired renal function (creatinine clearance 50–60 mL/min).

At the physician discretion, the S-1 schedule could be changed to 14 consecutive days followed by a 7-day rest at 3-week intervals, or to alternate days. Additionally, the dose could be reduced by 20 mg/day in patients with adverse effects. A reduction of 20 mg/day from the dosage calculated from the BSA was also allowed when patient had not recovered sufficiently from surgery, as evidenced by severely diminished physical strength, marked loss of body weight, and decreased food intake, compared with preoperative status. RDI was defined as the actual dose divided by the planned dose. Toxic effects were evaluated according to the Common Terminology Criteria for Adverse Events V4.0.

### 2.3. Measurement and assessment of psoas muscle volume

As described in a previous report, SYNAPSE VINCENT (Fujifilm, Tokyo, Japan) was used to calculate the total PMV (mm^3^) of each patient from preoperative computed tomography images.^[13]^ The total PMV (mm^3^) was then divided by the cube of height (m^3^) to produce normalized PMV values (mm^3^/m^3^). The optimal cutoff values for PMV were determined as 61.5 mm^3^/m^3^ for men and 44.1 mm^3^/m^3^ for women.^[13]^

### 2.4. Statistical analysis

Differences between the 2 groups were evaluated using the χ^2^ or Fisher exact probability test for categorical variables and the Mann–Whitney *U*-test for continuous variables. Survival curves were constructed using the Kaplan–Meier method. Differences between survival curves were examined by the log-rank or generalized Wilcoxon test. Receiver operating characteristic (ROC) analysis was used to determine the area under the curves (AUCs) of the RDI alone or a combination of the RDI and PMV. Univariate and multivariate analyses were performed using Cox proportional hazard regression models to identify factors with prognostic significance for OS. *P* < .05 was considered to denote statistical significance. All statistical analyses were performed using IBM SPSS Statistics for Windows (version 25; IBM, Armonk, NY).

## 3. Results

**Figure 1.**
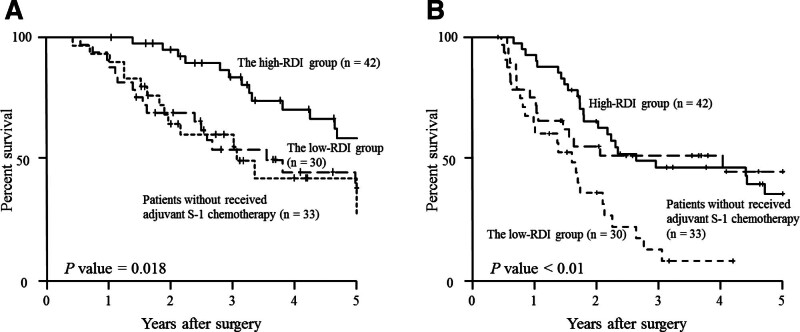
Overall survival curves according to RDI group (A); overall survival curves of patients who received sufficient RDI of adjuvant S-1 chemotherapy, insufficient RDI of adjuvant S-1 chemotherapy, and no adjuvant S-1 chemotherapy (B); and relapse-free survival curves according to RDI group (C) in patients who have undergone resection of pancreatic ductal adenocarcinoma. RDI = relative dose intensity.

**Figure 2.**
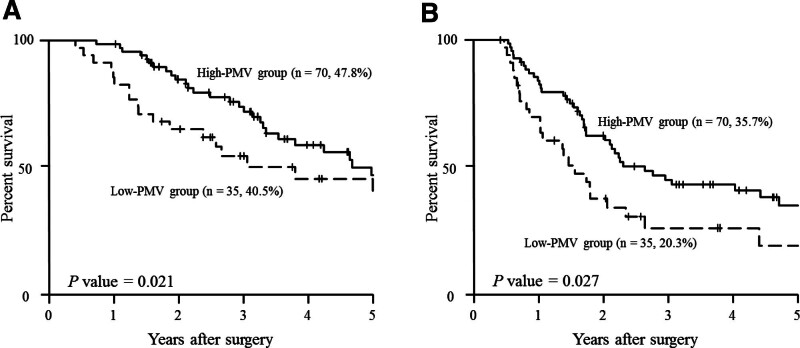
Overall survival curves according to PMV group (A) and relapse-free survival curves according to PMV group (B) in patients who have undergone resection of pancreatic ductal adenocarcinoma. PMV = psoas muscle mass volume.

S-1 was administered as postoperative adjuvant chemotherapy to 72 (68.6%) of the 105 patients in the current study; the remaining 33 did not receive adjuvant S-1 chemotherapy because they were older than 80 years or withheld consent. The mean RDI of the 72 patients who received adjuvant S-1 chemotherapy was 94.5% (range 2.7%–100.0%). The cutoff value of RDI for predicting the 3-year OS was determined at 95.6% by ROC analysis. The patients who received adjuvant S-1 chemotherapy were stratified into 2 groups according to this cutoff value: a high-RDI group (RDI ≥ 95.6%, n = 42) and a low-RDI group (RDI < 95.6%, n = 30). The patients who had not received adjuvant S-1 chemotherapy postoperatively were not included in either the high- or low-RDI group. Differences in clinicopathological characteristics between these 2 groups are shown in Table 1. We found a significant correlation between the high- and low-RDI groups regarding age: the patients were significantly older in the low- than the high-RDI group. Figure 1 shows the 5-year OS and RFS curves according to RDI. The 5-year OS rates in the high- and low-RDI groups were 59.1 % and 28.6%, respectively. This differences is significant (Fig. 1A, *P* = .018). Also, the 5-year OS rate of patients who received no adjuvant S-1 chemotherapy was 38.8 %, which was similar to that of patients in the low group and there was no significant difference in 5-year OS between patients who received an insufficient dosage of adjuvant S-1 chemotherapy and those who received no adjuvant S-1 chemotherapy. The 5-year RFS rate was significantly higher in the high- than in the low-RDI group (36.4% vs 9.2%, respectively; *P* < .01, Fig. 1B). According to the cutoff value of PMV, 105 patients were divided into high PMV (n = 60) or low PMV (n = 45). Table 2 shows relationship between clinicopathological characteristics and PMV. There were significant associations of PMV with age, sex and body mass index. Age was significantly younger in the high-PMV group than in the low-PMV group. Body mass index and ratio of men were significantly greater in the high-PMV group than in the low-PMV group. Both the 5-year OS and RFS rates were better in the high- than the low-PMV group (Fig. 2A and B). Multivariate analysis revealed that, in addition to lymph node metastasis and residual tumor, the RDI was an independent prognostic factor after resection of pancreatic cancer (Table 3).

**Table 1:** Comparison of clinicopathological characteristics between high relative dose intensity group and low relative dose intensity group of patients with pancreatic ductal adenocarcinoma who received adjuvant S-1 chemotherapy.

Characteristics	High-RDI group (n = 42)	Low-RDI group (n = 30)	*P* value
Age (yr), median (range)	69 (45–78)	72.5 (40–85)	.042
Sex (male), n (%)	21 (50.0%)	11 (36.7%)	.262
Body mass index (kg/m^2^), median (range)	23 (16.7–32.5)	22.3 (14.3–26.8)	.398
Tumor size (mm), median (range)	26 (9–75)	29 (1–85)	.548
Tumor location (pancreatic head), n (%)	28 (66.7%)	15 (50.0%)	.155
Histological grade (G1, %)	23 (54.8%)	20 (66.7%)	.310
Lymph node involvement (present), n (%)	24 (57.1%)	18 (60.0%)	.808
pStage, n (%)			.705
IA	8 (19.0%)	3 (10.0%)	
IB	8 (19.0%)	7 (23.3%)	
IIA	2 (4.8%)	2 (6.7%)	
IIB	18 (42.9%)	11 (36.7%)	
III	6 (14.3%)	7 (23.3%)	
Residual tumor (present), n (%)	2 (4.8%)	3 (10.0 %)	.643
ASA-PS (1 or 2, %)	38 (90.5%)	23 (76.7%)	.182
Preoperative CA19-9 (U/mL), median (range)	65.3 (1.0–4726.0)	89.4 (1.0–4017.0)	.824
Preoperative chemotherapy (present), n (%)	17 (40.5%)	11 (36.7%)	.744
Operative time (min), median (range)	497 (294–833)	483 (230–713)	.560
Estimated operative bleeding (mL), median (range)	417 (15–1930)	537 (10–2815)	.515
Albumin (g/dL), median (range)	4.1 (2.9–4.9)	4.3 (3.3–4.9)	.419
Prognostic nutritional index, median (range)	48.8 (37.3–56.8)	49.7 (37.4–67.0)	.850
Psoas muscle mass volume (mm^3^/m^3^), median (range)	57.1 (22.6–98.9)	56.3 (31.7–88.7)	.909

Continuous variables are expressed as the median and range.

ASA-PS = American Society of Anesthesiologists physical status, CA19-9 = carbohydrate antigen 19-9, G1 = well-differentiated, RDI = relative dose intensity.

**Table 2:** Comparison of clinicopathological characteristics between high psoas muscle volume group and low psoas muscle volume group of patients with pancreatic ductal adenocarcinoma.

Characteristics	High-PMV group (n = 70)	Low-PMV group (n = 35)	*P* value
Age (yr), median (range)	72 (40–85)	77 (55–89)	.026
Sex (male), n (%)	40 (57.1%)	12 (34.3%)	.027
Body mass index (kg/m^2^), median (range)	23 (15.6–32.5)	20.5 (14.0–28.5)	<.001
Tumor size (mm), median (range)	26 (1–85)	26 (9–48)	.583
Tumor location (pancreatic head), n (%)	40 (57.1%)	20 (57.1%)	1.000
Histological grade (G1, %)	41 (58.6%)	20 (57.1%)	.889
Lymph node involvement (present), n (%)	40 (57.1%)	17 (48.6%)	.406
pStage, n (%)			.813
IA	11 (15.7%)	8 (22.9%)	
IB	15 (21.4%)	9 (25.7%)	
IIA	4 (5.7%)	1 (2.9%)	
IIB	30 (42.9%)	13 (37.1%)	
III	10 (14.3%)	4 (11.4%)	
Residual tumor (present), n (%)	3 (4.3%)	3 (8.6 %)	.398
ASA-PS (1 or 2, %)	59 (84.3%)	27 (77.1%)	.370
Preoperative CA19-9 (U/mL), median (range)	61.5 (1.0–4726.0)	58.7 (1.0–1478.0)	.992
Preoperative chemotherapy (present), n (%)	25 (35.7%)	9 (25.7%)	.302
Operative time (min), median (range)	487 (230–833)	509 (219–780)	.527
Estimated operative bleeding (mL), median (range)	430 (1–2815)	535 (5–1805)	.448
Albumin (g/dL), median (range)	4.1 (2.9–4.9)	4.0 (3.0–4.7)	.251
Prognostic nutritional index, median (range)	49.3 (37.3–67.0)	47.4 (35.1–54.9)	.101

Continuous variables are expressed as the median and range.

ASA-PS = American Society of Anesthesiologists physical status, CA19-9 = carbohydrate antigen 19-9, G1 = well-differentiated, PMV = psoas muscle volume.

**Table 3:** Results of univariate and multivariate analyses of prognostic factors affecting overall survival in patients with pancreatic cancer who underwent S-1 adjuvant chemotherapy.

Variables	Univariate analysis			Multivariate analysis		
	HR	95% CI	*P*	HR	95% CI	*P*
Age (≥ 75 vs < 75)	1.863	0.856–4.054	.117			
Sex (male vs female)	0.614	0.287–1.313	.209			
Body mass index (≥ 22 kg/m^2^ vs. < 22 kg/m^2^)	0.968	0.458–2.047	.932			
Tumor size (≥ 20.0 mm vs < 20.0 mm)	1.063	0.452–2.504	.888			
Tumor location (body and tail head vs head)	2..058	0.979–4.329	.057			
Histological grade (G1 vs other)	0.840	0.399–1.768	.646			
Lymph node metastasis (present vs absent)	2.799	1.186–6.606	.019	2.852	1.199–6.783	.018
Residual tumor (present vs absent)	3.773	1.296–10.987	.015	3.334	1.120–9.920	.030
ASA-PS (3 vs 1 or 2)	1.993	0.752–5.285	.165			
Preoperative CA19-9 (≥ 139 U/mL vs < 139 U/mL)	0.582	0.221–1.533	.273			
Preoperative chemotherapy (present vs absent)	0.522	0.194–1.402	.197			
RDI (insufficient vs sufficient)	2.870	1.343–6.135	.007	3.255	1.489–7.117	.003
PMV (low vs high)	1.424	0.644–3.150	.382			

ASA-PS = American Society of Anesthesiologists physical status, CA19-9 = carbohydrate antigen 19-9, CI = confidence interval, G1 = well-differentiated, HR = hazard ratio, PMV = psoas muscle volume, RDI = relative dose intensity.

**Figure 3.**
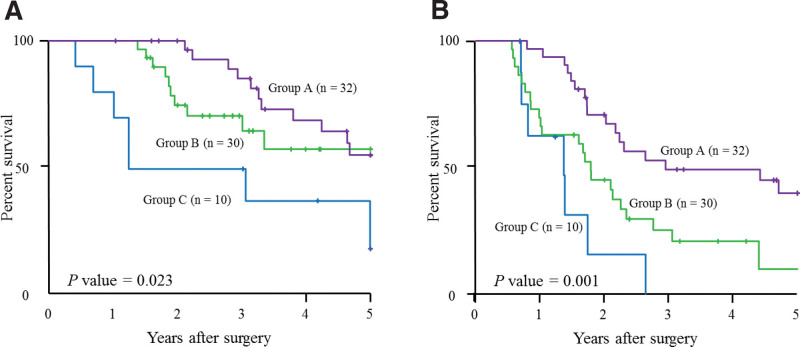
Survival curves according to combinations of RDI and PMV in patients who received adjuvant S-1 chemotherapy. Overall survival curves (A) and recurrence-free survival curves (B). PMV, psoas muscle mass volume; RDI, relative dose intensity Group A, patients with both high RDI and high PMV; Group B, patients with high RDI or high PMV (but not both); Group C, patients with both low RDI and low PMV. PMV = psoas muscle mass volume, RDI = relative dose intensity.

**Figure 4.**
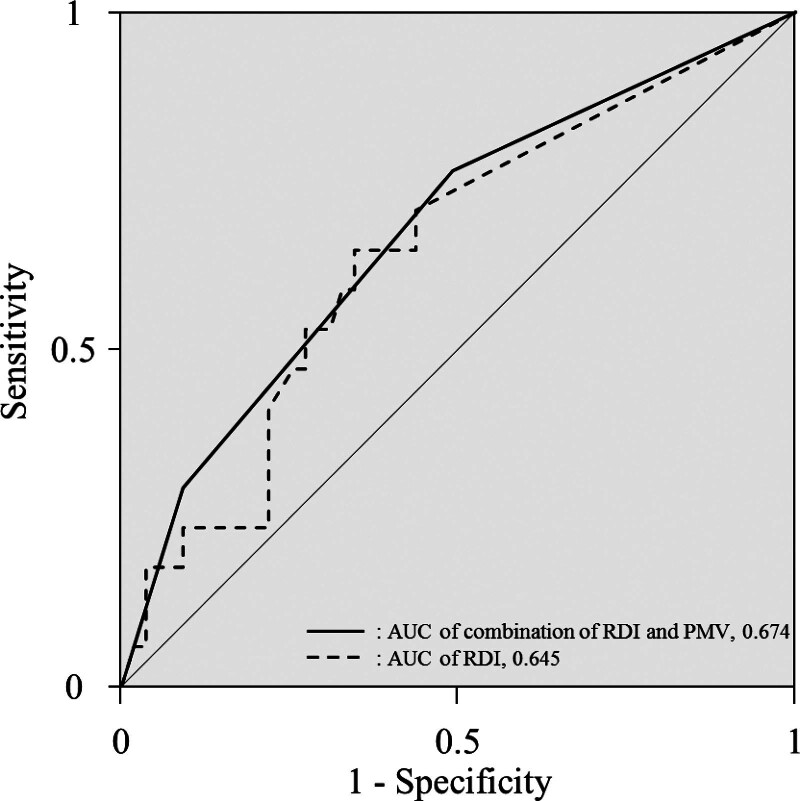
Area under the curves of RDI alone and combination of RDI and PMV for prediction of survival in patients who have undergone resection of pancreatic ductal adenocarcinoma. AUC = area under the curve, PMV = psoas muscle mass volume, RDI = relative dose intensity.

Furthermore, we stratified the patients who received adjuvant S-1 chemotherapy into 3 groups: A, patients with both high RDI and high PMV (n = 32); B, patients with high RDI or high PMV (but not both) (n = 30); and C, patients with both low RDI and low PMV (n = 10). Figure 3 shows the 5-year OS and RFS rates for the combination of the RDI and PMV. Statistically significant differences in 5-year OS and RFS were found between these 3 groups (5-year OS: *P* = .023, 5-year RFS: *P* = .001, Fig. 3A and B). The 5-year OS and RFS in each group were as follows: 55.4%, 40.6%, respectively, in the Group A; 57.6%, 10.9%, respectively, in the Group B; 18.8%, 0.0%, respectively, in the group C. ROC analysis produced the AUCs for predicting the 3-year OS for RDI alone and the combination of RDI and PMV, as shown in Figure 4. The AUC for the combination of RDI and PMV was 0.674, which is greater than that for RDI alone (0.645).

## 4. Discussion

In the present study, we found that patients who received an insufficient dosage of adjuvant S-1 chemotherapy had the same poor prognosis as those who received no adjuvant S-1 chemotherapy. Furthermore, the combination of RDI of adjuvant S-1 chemotherapy and PMV was superior to RDI alone for predicting survival after resection of PDAC.

Surgical resection is the only potentially curative treatment that can achieve long-term survival in patients with potentially resectable PDAC. However, even when curative resection with no residual cancer tissue has been achieved, the rate of recurrence is extremely high.^[4]^ Recurrence is attributed to the existence of micrometastases at the time of surgery.^[14,15]^ Hence, multidisciplinary treatment strategies, such as perioperative chemotherapy or chemoradiotherapy, are essential to improving survival of patients with PDAC. The JASPAC-01 trial found that postoperative adjuvant S-1 chemotherapy dramatically extends survival after resection of PDAC.^[9]^ Accordingly, adjuvant S-1 chemotherapy has become the standard treatment for such patients in Japan. In that study, doses of S-1 were reduced in 41% of patients who received this treatment and 68% of these patients discontinued treatment before completion of the protocol at the discretion of their physicians or after withdrawing their consent because of adverse events. Additionally, the survival of patients in cases where the dosage of S-1 was reduced and in those where it was not reduced was not evaluated. Only a few studies have reported on the important impact on survival of both completion of planned postoperative adjuvant chemotherapy and of dose intensity after surgery for PADC.^[16–18]^ Kobayashi et al found an association between a low total dose intensity of adjuvant S-1 chemotherapy and a poorer prognosis in patients who had undergone pancreatectomy for pancreatic cancer.^[18]^ However, their cohort included approximately 30% of patients with invasive IPMC and also included patients having developed early recurrences during administration of adjuvant S-1 chemotherapy. Furthermore, Yabusuki et al reported that a decrease of RDI of adjuvant chemotherapy was significantly associated with poor prognosis in patients with PADC.^[16]^ Although patients who received adjuvant S-1 chemotherapy with high RDI had longer survival than those with low RDI in sub-analyses, this was a small study; only 49 patients received adjuvant S-1 chemotherapy. Therefore, the prognostic significance of the RDI of adjuvant S-1 chemotherapy after resection of PDAC remains unclear thus far. Our finding that administering sufficient S-1 dose intensity is important in achieving favorable outcomes after resection of PDAC is therefore very significant.

A close relationship has been established between skeletal muscle mass and tolerance to adjuvant chemotherapy. Lower muscle mass is associated with greater toxicity and poorer adherence to postoperative adjuvant chemotherapy for colon and breast cancer.^[19,20]^ Furthermore, a decrease in RDI of adjuvant chemotherapy is reportedly related to skeletal muscle mass index in patients with colorectal cancer.^[21]^ However, in the current study, we did not find a significant correlation between dosage of adjuvant S-1 chemotherapy and PMV in patients who had undergone resection of PDAC. However, we did find that patients with low PMV had significantly shorter survival than those with high PMV. Skeletal muscle loss is associated with impairment of immune responses in patients with cancer because counts of immunocytes pooled in skeletal muscle decreased.^[22]^ This may explain the prognostic relationship between skeletal muscle mass and advanced cancer. Therefore, a low PMV may reflect more advanced PDAC. We thus hypothesized that the combination of RDI and PMV might better predict prognosis after resection of PDAC than RDI alone. Indeed, we found that the AUC of the combination of RDI and PMV for predicting OS was greater than the AUC of RDI alone. Finally, the prognosis of study patients with both low RDI and low PMV was worse than that of those with other combinations of RDI and PMV.

This study had several limitations. First, it was a retrospective single institution study with a small patient cohort, which may have produced some bias and limited the generalizability of the findings. Second, it may not have been valid to exclude patients whose disease recurred within 6 months of surgery. Third, our patients had received a variety of chemotherapy regimens preoperatively, which may have influenced eventual outcomes. Our findings need to be verified in a larger, prospective, multicenter study.

In conclusion, we found that a sufficient dosage of adjuvant S-1 chemotherapy is important in improving the OS after resection of PADC in this study. In addition, the combination of RDI and PMV may be a more useful predictor of the prognosis than RDI alone after resection of PDAC: patients with an insufficient dosage of adjuvant S-1 chemotherapy and low skeletal muscle mass volume have worse prognoses than those with other combinations of these variables.

## Acknowledgments

We thank Dr Trish Reynolds, MBBS, FRACP, from Edanz (https://jp.edanz.com/ac) for editing a draft of this manuscript.

## Author contributions

**Conceptualization:** Teruhisa Sakamoto, Kyoichi Kihara.

**Data curation:** Yuki Murakami, Kyoichi Kihara.

**Formal analysis:** Mikiya Kishino, Yuki Murakami, Kozo Miyatani, Yuji Shishido, Manabu Yamamoto.

**Investigation:** Mikiya Kishino, Yuki Murakami, Kozo Miyatani, Yuji Shishido.

**Methodology:** Takehiko Hanaki, Tomoyuki Matsunaga.

**Software:** Kozo Miyatani.

**Writing – original draft:** Teruhisa Sakamoto.

**Writing – review & editing:** Naruo Tokuyasu, Yoshiyuki Fujiwara.
